# Identification and management of Lynch syndrome in the Middle East and North African countries: outcome of a survey in 12 countries

**DOI:** 10.1007/s10689-020-00211-3

**Published:** 2020-10-24

**Authors:** Mohammad Sina, Zeinab Ghorbanoghli, Amal Abedrabbo, Fahd Al-Mulla, Rihab Ben Sghaier, Marie-Pierre Buisine, George Cortas, Ladan Goshayeshi, Andreas Hadjisavvas, Wail Hammoudeh, Waseem Hamoudi, Carol Jabari, Maria A. Loizidou, Keivan Majidzadeh-A, Makia J. Marafie, Gurbankhan Muslumov, Laila Rifai, Rania Abu Seir, Suzan M. Talaat, Berrin Tunca, Hadia Ziada-Bouchaar, Mary E. Velthuizen, Ala I. Sharara, Aysel Ahadova, Demetra Georgiou, Hans F. A. Vasen, on behalf of the Middle East Network on Hereditary Colorectal Cancer (HCCN-ME)

**Affiliations:** 1 Genetics Department, Breast Cancer Research Center, Motamed Cancer Institute, ACECR, Tehran, Iran; 2 A. Nocivelli Institute for Molecular Medicine, Department of Molecular and Translational Medicine, University of Brescia, 25123 Brescia, Italy; 3 Department of Gastroenterology and Hepatology, Leiden University Medical Center, Albinusdreef 2, 2333 ZA Leiden, The Netherlands; 4 Dutch Hereditary Cancer Registry, Leiden, The Netherlands; 5 Department of Pediatrics, Makassed Islamic Charitable Hospital, Jerusalem, Palestine; 6 Department of Genetics and Bioinformatics, Dasman Diabetes Institute, P.O. Box 1180, 15462 Dasman, Kuwait; 7 Cytogenetic, Molecular Genetics and Human Reproduction Biology - Farhat, HACHED Hospital, Sousse, Tunisia; 8 Unit of Molecular Oncology and Genetics, Institute of Biochemistry and Molecular Biology, Lille University Hospital, Lille, France; 9 Department of Gastroenterology, St. George Hospital Medical Center, University of Balamand Medical School, Beirut, Lebanon; 10 Department of Gastroenterology and Hepatology, Faculty of Medicine, Mashhad University of Medical Sciences, Mashhad, Iran; 11 Department of Electron Microscopy/Molecular Pathology, The Cyprus Institute of Neurology and Genetics, Nicosia, Cyprus; 12 Department of Internal Medicine, Arabcare Hospital, Ramallah, Palestine; 13 Department of Gastroenterology, The Royal Hospital, Amman, Jordan; 14 Patient’s Friends Society, Jerusalem, Palestine; 15 Hebron University, Hebron, Palestine; 16 Kuwait Medical Genetics Centre, Maternity Hospital, 13059 Safat, Kuwait; 17 Colorectal Surgery Department, Scientific Center of Surgery, Baku, Azerbaijan; 18 Centre Hospitalier Universitaire IBN SINA, Rabat Instituts, Institut National D’Oncologie Sidi Mohamed Ben Abdellah, BP 6213, Rabat, Maroc; 19 Al-Quds University, Abu-Dis, Palestine; 20 Ahmed Maher Teaching Hospital, Cairo, Egypt; 21 Department of Medical Biology, Medical Faculty, Uludag University, Bursa, Turkey; 22 Laboratory of Biology and Molecular Genetics, Faculty of Medicine, University 3, Rabah Bitat, Constantine, Algeria; 23 Department of Genetics, University Medical Center Utrecht (Location WKZ), Utrecht, the Netherlands; 24 Division of Gastroenterology, American University of Beirut Medical Centre, Beirut, Lebanon; 25 Department of Applied Tumour Biology, Institute of Pathology, University Hospital Heidelberg, Heidelberg, Germany; 26 Cooperation Unit Applied Tumour Biology, German Cancer Research Center (DKFZ), Heidelberg, Germany; 27 Department of Clinical Genetics, London North West University Healthcare, London, UK

**Keywords:** Colorectal cancer, Lynch syndrome, Middle Eastern countries, North African countries

## Abstract

**Background:**

Lynch syndrome (LS), the most common inherited form of colorectal cancer (CRC), is responsible for 3% of all cases of CRC. LS is caused by a mismatch repair gene defect and is characterized by a high risk for CRC, endometrial cancer and several other cancers. Identification of LS is of utmost importance because colonoscopic surveillance substantially improves a patient’s prognosis. Recently, a network of physicians in Middle Eastern and North African (ME/NA) countries was established to improve the identification and management of LS families. The aim of the present survey was to evaluate current healthcare for families with LS in this region.

**Methods:**

A questionnaire was developed that addressed the following issues: availability of clinical management guidelines for LS; attention paid to family history of cancer; availability of genetic services for identification and diagnosis of LS; and assessment of knowledge of LS surveillance. Members of the network and authors of recent papers on LS from ME/NA and neighbouring countries were invited to participate in the survey and complete the online questionnaire.

**Results:**

A total of 55 individuals were invited and 19 respondents from twelve countries including Algeria, Azerbaijan, Cyprus, Egypt, Iran, Jordan, Kuwait, Lebanon, Morocco, Palestine, Tunisia, and Turkey completed the questionnaire. The results showed that family history of CRC is considered in less than half of the surveyed countries. Guidelines for the management of LS are available in three out of twelve countries. The identification and selection of families for genetic testing were based on clinical criteria (Amsterdam criteria II or Revised Bethesda criteria) in most countries, and only one country performed universal screening. In most of the surveyed countries genetic services were available in few hospitals or only in a research setting. However, surveillance of LS families was offered in the majority of countries and most frequently consisted of regular colonoscopy.

**Conclusion:**

The identification and management of LS in ME/NA countries are suboptimal and as a result most LS families in the region remain undetected. Future efforts should focus on increasing awareness of LS amongst both the general population and doctors, and on the improvement of the infrastructure in these countries.

**Electronic supplementary material:**

The online version of this article (10.1007/s10689-020-00211-3) contains supplementary material, which is available to authorized users.

## Introduction

Colorectal cancer (CRC) is the third most commonly diagnosed cancer worldwide and the fourth leading cause of cancer-related death [1]. Although the incidence of CRC in Middle Eastern and North African (ME/NA) countries is low compared to Western countries [2], recent reports suggest that it is increasing rapidly [3].

One option for prevention of CRC is offering surveillance to individuals at high risk of inherited CRC. The most common hereditary form of CRC is Lynch syndrome (LS), which is responsible for 3% of all cases of CRC [4]. LS is an autosomal dominant disorder caused by a pathogenic variant in one of the mismatch repair (MMR) genes, which include *MLH1, MSH2 (EPCAM), MSH6* and *PMS2*. Loss of MMR function leads to the molecular phenotype of microsatellite instability (MSI) in tumours, which in turn drives carcinogenesis [4].

LS is characterized by a very high risk of developing CRC and endometrial cancer. The risk of developing a metachronous CRC is also high in Lynch syndrome individuals, therefore such patients should be offered a more extensive surgical treatment compared to sporadic CRC patients. LS families also show other cancers including cancers of the urinary tract, ovaries, stomach, pancreas, biliary tract, skin and cancers of the brain. Cancer risks and the spectrum of tumours depends on the type of underlying pathogenic variant [5]. The molecular phenotype of MSI characteristic for LS-associated tumours can be identified by PCR fragment length analysis of microsatellite markers or immunohistochemical (IHC) analysis of the MMR proteins. The diagnosis is confirmed by analysis of germline DNA.

Several studies have demonstrated that periodic examination of the colon leads to an improved prognosis and a substantial decrease in mortality [6, 7]. Identification of LS and the participation of families in surveillance programs is therefore of paramount importance.

In 2017, at a conference of the Palestinian Society of Gastroenterology in Jericho, a new network of doctors interested in hereditary CRC in ME/NA countries was established, now referred to as the Hereditary Colorectal Cancer Network-Middle East (HCCN-ME) [8]. The main goal of this network is to improve care for individuals with inherited CRC in these countries. In order to reach this goal, we first need a picture of current management of LS in the region. Thus, the aim of the present study was to survey the state of LS healthcare in ME/NA countries.

## Methods

Our first task was to develop a questionnaire that addresses the most relevant issues (Supplementary file 1).The questionnaire contains three categories of questions: (1) general questions about the availability of guidelines for diagnosis and management of LS in each country, including questions concerning the attention paid by physicians to family histories of CRC (cancer), (2) questions about the genetic services available for the identification and diagnosis of LS, and (3) questions that assess general knowledge of the clinical management of LS patients.

All members of the network, and authors of recent articles on LS from ME/NA or neighbouring countries, were invited to participate in the survey. The invitation mail contained a link to the online questionnaire (developed using SurveyMonkey). The respondents came from 12 countries in the ME/NA region.

## Results

A total of 22 (40%) out of 55 professionals invited to participate in the survey responded. Three participants were excluded because they completed too few questions. The remaining 19 respondents were from twelve countries, including Algeria, Azerbaijan, Cyprus (two respondents), Egypt, Iran (two respondents), Jordan, Kuwait (two respondents), Lebanon, Morocco, Palestine (four respondents), Tunisia (two respondents), and Turkey (Table 1).

**Table 1:** Countries involved in the survey, including population (millions) (https://data.worldbank.org/)

Countries (Population)
Kuwait (4,137,309)
Iran (81,800,269)
Turkey (82,319,724)
Azerbaijan (9,942,334)
Lebanon (6,848,925)
Palestine (4,862,979)
Jordan (9,956,011)
Egypt (98,423,595)
Morocco (36,029,138)
Tunisia (11,565,204)
Cyprus (1,189,265)
Algeria (42,228,429)
**All countries (389,303,182)**

The respondents included eight clinical/molecular geneticists, four gastroenterologists, one surgeon, one researcher, one specialized nurse, one paediatrician, two biological scientists and one pathologist. Twelve of the 19 respondents were affiliated to a University Medical Centre.

The survey revealed that guidelines for the diagnosis and management of LS were available in three countries. In two countries, the respondents were unaware of clinical guidelines in their country. In five countries, most doctors pay appropriate attention to family history of CRC.

Genetic services were available in a research setting in one country, in only a few hospitals in six countries, in several hospitals in three countries and were absent in two countries. The most common explanation for the limited availability of these services was lack of funds in five countries, lack of interest or knowledge in four countries and a lack of trained geneticists in one country.

Where genetic services were available, pedigree analysis was offered in ten countries, genetic counselling in eight countries, IHC or MSI-analysis in eight countries, MLH1-promotor methylation or BRAF-analysis of tumours (performed to exclude methylation of the MLH1-promotor as cause of loss of MMR-function) in eight countries and (Sanger) sequencing or Next Generation Sequencing (NGS) in ten countries.

Clinical criteria were used for the identification of LS in eight of the 12 countries, while systematic screening of tumours was offered in only one country (i.e., Cyprus). Respondents from almost all countries (11 out of 12) stated that most LS families go unidentified in their country. By contrast, the respondent from Turkey reported that most LS patients in that country were identified.

With respect to the management of LS, colonoscopic surveillance is offered in 11 out of 12 countries and surveillance of the endometrium in five of the 12 countries. As regards the less common cancers observed in LS, surveillance of the urinary tract is offered in three countries and upper GI tract in five countries. Assessment of H.pylori infection was performed in four of the 12 countries. Extended surgery (subtotal colectomy) for CRC diagnosed at < 50–60 years was an option in six of the 12 countries. A registry of families with Lynch syndrome was available in seven out of the 12 countries. The replies to five questions have not been shown because most respondents did not have information about the issue that was addressed. The outcome of the survey is summarized in Table 2.

**Table 2:** Main outcomes from survey on current Lynch syndrome healthcare in the Middle East, North African and neighbouring countries

	Countries (total 12)
1. Guidelines available for management of LS?	3
2. Appropriate attention to family history of CRC by most doctors?	5
3. Genetic services available:	
Several hospitals	3
Few hospitals	6
Only in research setting	1
Not available	2
4. Explanations for limited genetic services?	
Lack of finances	5
Lack of interest/knowledge	4
Lack of geneticists/genetic counsellors	1
5. Strategies for identification of LS?	
Clinical criteria, i.e., Amsterdam II criteria or Revised Bethesda guidelines	8
Universal screening of all new CRC or endometrial cancer	1
6. Are most LS families identified?	
Most not identified	11
Most identified	1
7. Is surveillance offered?	
Colonoscopic surveillance?	11
Endometrial surveillance?	5
Urinary tract?	3
Upper GI tract?	5
Helicobacter Pylori assessment?	4
8. Surgical treatment:	
Subtotal colectomy offered to patients with CRC < 50 years	6
9. Lynch syndrome Registry available?	7

## Discussion

Lynch syndrome is an example of an inherited form of cancer for which surveillance and early treatment is extremely effective [6, 7]. It is one of the most common inherited forms of cancers, affecting approximately 1/300 people in Western countries [9]. Although data on the incidence of LS in Middle East and North African (ME/NA) countries are limited, studies suggest that LS accounts for a similar proportion of CRC compared to Western countries [10]. Based on these figures and an estimated population of 390 million in the 12 countries covered in the survey, we estimate that more than one million individuals in these countries have LS.

The current survey is the first study to provide information on prevailing LS healthcare in the ME/NA countries. The survey revealed that a family history of CRC receives appropriate attention in fewer than half the countries, and guidelines for the management of LS are available in only three of the 12 countries surveyed. The identification and selection of families for further genetic testing were generally based on clinical criteria, i.e., the Amsterdam criteria II or Revised Bethesda criteria, but universal screening was performed in one country. Genetic services are limited to only a few hospitals or a research setting in most countries. The surveillance offered in the majority of countries consists of regular colonoscopy, 1x/2 years, from age 20–25 years, while screening of the endometrium is available in less than half of the countries surveyed. In seven countries, a Hereditary Cancer Registry is available.

Ten years ago, a similar survey based on 30 respondents was performed in 14 European countries [11]. The results indicated that most countries devoted sufficient attention to a family history of CRC, although the quality of the obtained family history was considered suboptimal in some countries. Guidelines for the management of LS were available in most of the countries surveyed at that time. In addition, clinical criteria were mainly used for the selection of families for genetic testing (in all but one country), which is similar to the current survey. Nowadays, universal screening of all newly detected cases of CRC and endometrial cancer is recommended in the national guidelines of most European countries.

Another comprehensive survey of worldwide patterns of practice in the diagnosis and management of Lynch syndrome was performed recently by the International Mismatch Repair Consortium (IMRC)[12]. Data were collected from institutions in 21 countries (55 respondents) in Europe, North, Central and South America, Asia and Australasia. ME/NA countries were not included. Fifty-five percent of the respondents reported routine screening of newly identified LS-related cancers, and 27% reported relying on clinical criteria together with selective tumour testing and germline analysis. Most institutions (64%) also reported the use of multigene panels. Reported risk management practices included 1–2 yearly colonoscopy in almost all programs (98%) and gynaecological screening in 78%. Gastric cancer screening was recommended in 56% of programs, especially so in Asia, North, Central and South America. The authors concluded that there is widespread heterogeneity in management practices for LS worldwide, which is probably due to the rapid pace of emerging technology and regional differences in resources.

A major strength of the survey is the large number of participating countries which taken together represent a population of 390 million people (Table 1). A concurrent limitation was the relatively low number of respondents from each country, which is probably attributable to a lack of awareness or interest in LS in these countries.

What are the implications of the results of this survey? In view of the finding that most families with LS in ME/NA countries probably remain unidentified, the first and foremost question is how the recognition of these families might be improved and which tools should be used. In this respect, we must recognize that the economic outlook is poor in most of the included countries and therefore the best approach will likely vary between countries and depend on the available (financial) resources. Regardless of the economic situation, obtaining a detailed family history of (colorectal) cancer could be easily implemented in all countries. A detailed family history requires asking whether cancer occurs in first- and second-degree relatives, including the type of cancer and the age at diagnosis [11, 13]. Online risk assessment tools can be used to assist with risk stratification and the management of patient and family screening [13]. In countries with limited financial resources, clinical criteria such as the revised Bethesda criteria can be used to select families for molecular testing of tumours, which is usually based on IHC analysis of the MMR proteins. In these countries, obtaining an appropriate family history is particularly important as it is essential to determine whether the clinical criteria have been met. In countries with sufficient financial resources, universal screening with IHC testing of all newly detected CRC and endometrial cancer below age 70 (or independent of age) is probably the best approach [14], although the cost-effectiveness of this approach in ME/NA countries remains to be determined.

For successful implementation of tumour analysis and subsequent germline analysis, a well-established infrastructure is needed. One of the reasons why genetic services are limited is that clinical geneticists or genetic counsellors are scarce in many countries. In view of the remarkable progress of genetic testing across a wide area of medicine, training of more geneticists and genetic counsellors should be promoted in all ME/NA countries and more attention should be paid to genetics in all medical curricula. In the interim, training of current healthcare professionals regarding hereditary cancer and genetic counselling should be organized, for example via e-learning modules [15]. In addition, an online forum could further assist the establishment of infrastructure through the use of shared resources as well as forming a supportive professional network to share experiences and opinions.

Registries of families with inherited cancer should also be established, with the aim of encouraging participation in prevention programs and to guarantee the continuity of lifetime surveillance programs [16].

Another key issue is the need to increase awareness of the importance of hereditary factors in the development of cancer amongst the general population as well as doctors. Information pamphlets for patients with (colorectal) cancer should contain a paragraph about hereditary cancer and the need for preventative measures in case of confirmed hereditary cancer. This information should also be provided on hospital websites accessible to the general public. To help improve knowledge about hereditary cancer among physicians in ME/NA countries, all major scientific conferences concerning CRC should include sessions on hereditary CRC, including polyposis and Lynch syndrome. In addition, guidelines should be developed that correspond to the specific situation in a country.

A further suggestion to help raise awareness of hereditary conditions among the public and healthcare professionals is the engagement of charities and non-profit governmental organisations to support and promote policy change. These organisations can also aid in the development of screening programmes, as well as universal genetic screening strategies for Lynch syndrome.

In conclusion, the current study shows that the identification and management of LS in ME/NA countries is still suboptimal. Future efforts should focus on increasing awareness of LS amongst both the general population and doctors, and on improvement of the infrastructure in these countries. A summary of our recommendations to improve LS care can be found in Table 3.

**Table 3:** How to improve the identification and management of LS

More attention should be paid to obtaining a family history of cancer (CRC)	
Development of online family history assessment tools to aid identification of individuals at high risk [13]	
The medical curriculum of doctors should be expanded to include cancer geneticists	
Training of more genetic consultants and counsellors who can assist in further training of cancer clinicians in their respective countries (train the trainer)	
Genetic services should be made more widely available, starting with a few referral centres and subsequently expanding to more centres, depending on the size of the country	
Guidelines for diagnosis and management should be developed that correspond to the specific (financial) resources of each country	
National or regional registries of LS families should be established in all countries with the aim of improving participation in surveillance programs and to guarantee the continuity of lifelong surveillance programs	
Major gastroenterology meetings in the Middle East and North Africa should include sessions on hereditary CRC	
Provision of online genomics education courses to currently practicing physicians [15]	
Engagement with government/charity sector in these countries to increase awareness	
Improvement of available online information (www.HCCN-ME.com) in a variety of languages so that patients can learn more about hereditary cancer and enquire about their family history	
Introduce the use of Health Information Systems that necessitate the inclusion of systematic data	

## Electronic supplementary material

Below is the link to the electronic supplementary material.

Supplementary file1 (PDF 106 kb)
