# Supplementary material for: Identification and management of Lynch syndrome in the Middle East and North African countries: outcome of a survey in 12 countries
Source: Fam Cancer. 2020 Oct 24;20(3):215–21. doi: 10.1007/s10689-020-00211-3 (PMC8214581; doi:10.1007/s10689-020-00211-3)
Supplement: Supplementary file 1 — Supplementary file1 (PDF 106 kb) [file 10689_2020_211_MOESM1_ESM.pdf]

Assessment of current health care Lynch syndrome in the Middle East and North Africa-2019

A. Background

1. What is your medical specialty?

- ○ Clinical genetics
- ○ Gastroenterology
- ○ Surgery
- ○ Oncology
- ○ Other clinical specialty; please specify
- ○ Gynaecology
- ○ Pathology
- ○ Pediatrician

2. Which of the following statements best depict your clinical setting?

- ○ Academic/University setting
- ○ Public hospital
- ○ Private hospital
- ○ Private practice
- ○ Other; please. Specify:

3. Years of experience in your profession?

years

Assessment of current health care Lynch syndrome in the Middle East and North Africa-2019

B. Guidelines, family history, available services and technology

4. Are guidelines on the identification and management of Lynch syndrome available in your country?

- ◯ Yes
- ◯ No
- ◯ Don’t know

5. Do physicians or specialists in your country generally pay attention to family history for cancer?

- ◯ Yes, in all patients
- ◯ Only in patients with cancer
- ◯ Only a few doctors pay attention to family history for cancer
- ◯ Don’t know
- ◯ Other; please specify

6. Are genetic services (for example, Department of Clinical Genetics in Hospital) currently available in your country?

- ◯ Yes, in several hospitals
- ◯ Yes, but only in a few hospitals
- ◯ Only available in research setting
- ◯ Other; please specify
- ◯ No
- ◯ Don’t know

7. If genetic services are not available, who is currently responsible for pedigree analysis and genetic counselling?

- ○ Clinician (Oncologist, Gastroenterologist, Surgeon)
- ○ Pedigree analysis and genetic counselling are currently not offered
- ○ Don’t know
- ○ Not applicable

8. If genetic services are not available, what are the reasons?

- ☐ Lack of knowledge
- ☐ Lack of finances
- ☐ Lack of interest
- ☐ Not applicable
- ☐ Other, please specify

9. If genetic services are available, which specific services are available?

- ☐ Pedigree analysis
- ☐ Genetic counselling
- ☐ Immunohistochemical (IHC) analysis of mismatch repair proteins in colorectal cancer
- ☐ MSI-analysis of colorectal cancer
- ☐ MLH1-promotor methylation test
- ☐ BRAF-analysis
- ☐ Sanger sequencing
- ☐ NGS panel testing
- ☐ Don’t know
- ☐ Other, please specify:

10. If genetic testing is available, who covers the costs?

- ☐ Patient’s insurance
- ☐ Government
- ☐ Patients themselves
- ☐ Don’t know
- ☐ Other, please specify

C. Identification of Lynch syndrome families

11. How are Lynch syndrome families currently being identified in your country?

- [ ] Family history showing multiple (3 or more) cases of colorectal cancer (CRC)
- [ ] Clinical criteria: Amsterdam criteria II (3 relatives with CRC, one relative is first degree relative of other two, in at least two successive generations, one diagnosed<50 yrs)
- [ ] Clinical criteria: Revised Bethesda criteria (CRC<50 yrs, personal history of multiple CRC, 3 first or second degree relatives with CRC in family)
- [ ] Systematic screening of all CRC <70 yrs using IHC- or MSI-analysis
- [ ] Systematic screening of all endometrial cancer <60 yrs using IHC- or MSI-analysis
- [ ] No efforts are undertaken in my country to identify Lynch syndrome
- [ ] Don’t know
- [ ] Other, please specify

12. Do you feel that most Lynch syndrome families are currently identified in your country?

- ○ Yes, most families are identified
- ○ Only few families are identified
- ○ Most families are not identified
- ○ Don’t know
- ○ Other, please specify

Assessment of current health care Lynch syndrome in the Middle East and North Africa-2019

D. Clinical Management

13. Do you discuss the option of extended surgical resection (subtotal colectomy) in patients highly suspected of Lynch syndrome with a newly diagnosed (early) CRC?

- ◯ Yes in patients with CRC <50 yrs
- ◯ Yes in patients with CRC <60 yrs
- ◯ No
- ◯ Don’t know

14. Do you offer monotherapy with 5 Fluorouracil (5FU) in patients with a mismatch repair deficient CRC (Lynch syndrome or sporadic mismatch repair deficient CRC)?

- ◯ No, because studies showed that such therapy is not effective
- ◯ Yes
- ◯ Don’t know

15. Do you offer patients with an advanced/metastatic mismatch repair deficient CRC, treatment with PDL1 (checkpoints) inhibitors?

- ◯ Yes
- ◯ No, drug is not available
- ◯ Don’t know

Assessment of current health care Lynch syndrome in the Middle East and North Africa-2019

E. Surveillance/prophylactic surgery

16. Do you offer surveillance to individuals with suspected or proven Lynch syndrome?

- ◯ Yes
- ◯ No
- ◯ Don’t know

17. Which surveillance program do you offer?

- ☐ Surveillance colonoscopy: 1x/2 yrs, from age 20-25 yrs
- ☐ Gynaecological examination including transvaginal ultrasound and/or pipelle curettage?
- ☐ Urinalysis and/or urine cytology; 1x/1-2 yrs
- ☐ Upper GI-endosopy; 1x/1-2 yrs
- ☐ Assessment H.Pylori infection and, if positive, eradication
- ☐ Other surveillance procedures; please, specify

18. Do you offer prophylactic hysterectomy and bilateral oophorectomy by the age of 40 yrs in patients with proven Lynch syndrome and a complete family?

- ◯ Yes
- ◯ No
- ◯ Don’t know

19. Do you have a registry in your country that encourages high risk individuals to participate in a surveillance program, collects results of surveillance examinations and ensures long-term follow-up?

- ◯ Yes
- ◯ Yes, just started
- ◯ No
- ◯ Don’t know

Assessment of current health care Lynch syndrome in the Middle East and North Africa-2019

F. Other issues

20. Would you be interested to participate in a Familial CRC Conference organized by the Middle East network?

- ◯ Yes, please send me an invitation
- ◯ May be in the future
- ◯ No

21. Would you like to become a member of the Middle East Hereditary Colorectal Cancer Network?

- ◯ Yes
- ◯ Maybe in the future
- ◯ No

22. personal information

Name: ____________________

Affiliation: ____________________

Place: ____________________

Country: ____________________

Email Address: ____________________
